# Enhanced semiempirical QM methods for biomolecular interactions

**DOI:** 10.1016/j.csbj.2015.02.004

**Published:** 2015-02-28

**Authors:** Nusret Duygu Yilmazer, Martin Korth

**Affiliations:** Institute of Theoretical Chemistry, Ulm University, D-89069 Ulm, Germany

**Keywords:** Computational chemistry, Biomolecular interactions, Semi-empirical QM methods, Dispersion interactions, Hydrogen-bond-interactions

## Abstract

Recent successes and failures of the application of ‘enhanced’ semiempirical QM (SQM) methods are reviewed in the light of the benefits and backdraws of adding dispersion (D) and hydrogen-bond (H) correction terms. We find that the accuracy of SQM-DH methods for non-covalent interactions is very often reported to be comparable to dispersion-corrected density functional theory (DFT-D), while computation times are about three orders of magnitude lower. SQM-DH methods thus open up a possibility to simulate realistically large model systems for problems both in life and materials science with comparably high accuracy.

## 1 Introduction

Recent years have seen a renaissance of semi-empirical quantum mechanical (SQM) electronic structure theory methods. This is mainly due to the fact that they still provide ‘unprecedented bang for the buck’, as Tim Clark has once put it [1]. Because computer scientists and engineers have again provided theoretical chemists with largely increased computer power over the last decade, realistic model systems for biomacromolecules and nanostructures have now come into the reach of SQM (but not yet DFT) methods. A number of recent successes in the application of SQM methods in these fields (see below for details) have also made people more aware of the ‘SQM gap’ between classical molecular mechanics (MM) and fully quantum mechanical (QM) wave function theory (WFT) methods like MP2 and density function theory (DFT) approaches: The difference of about six orders magnitudes between the typical run times of MM and DFT calculations is so large that there is by far enough space for an intermediate level of theory in between these two. And indeed, SQM calculations are roughly three orders of magnitude faster than DFT calculations, but still three orders of magnitude slower than MM ones: Whenever a system is in the reach of DFT SQM methods will allow fast (pre-)optimization, dynamical studies and high-throughput-screening based *in silico* optimization of this system. Nowadays, ‘linear scaling’ approaches have made the DFT treatment of large biomolecular systems possible [2]; similar techniques are of course available for SQM (e.g. MOZYME in MOPAC). The biggest advantages of SQM methods are thus their speed in comparison to DFT and versatility in comparison to MM methods. Very accurate structures and energies are obtained for ‘standard’ organic chemistry [3]. The often comparably simple electronic interactions in biomolecules and non-covalent interaction dominated nano-systems (as opposed to for instance heterogeneous catalysis) makes SQM methods especially useful also for work in life science and in some fields of materials science. For the latter two application fields, an accurate treatment of non-covalent interactions is absolutely essential, which is why it seems fair to say that ‘enhanced’ (empirically corrected) SQM methods of the SQM-DH type have contributed substantially to the above mentioned renaissance of SQM methods.

The accurate modeling of non-covalent interactions is indeed problematic for ‘pure’ SQM methods [4]. Unlike standard MM methods [5,6], SQM can take charge transfer and polarization effects into account, but results for dispersion and hydrogen-bond interactions are not accurate enough [1,7–9], though several examples of successful biomolecular simulation with ‘pure’ SQM methods exist [10–18], sometimes with some corrections for hydrogen-bond interactions [1,9,19–23]. It is noteworthy that most standard MM methods also have substantial problems with hydrogen-bonds [24], but dedicated (three or more body) hydrogen correction terms are usually too costly for the typical biomolecular force field application scenarios, e.g. dynamical studies of large biologically relevant molecular systems. We have reviewed the historical development and technical details of SQM-DH methods before [25], so that here we will take the opportunity to focus on a discussion of the recent successes and failures of the application of these approaches after a very short overview of the basics in Section 2. Section 3 is devoted to the development and benchmarking of SQM-DH methods, Section 4 gives on overview of studies using these methods. Two paragraphs at the of Section 4 are dedicated to applications in materials science and in general chemistry, before a summary of the main findings and an outlook are given in Section 5.

## 2 Empirical correction terms

SQM-DH methods, with empirical MM-type correction terms for either or both dispersion (D) and hydrogen-bond (H) interactions have been introduced only rather recently. The D terms are the same as for DFT-D methods:

(1) $$E_{\mathrm{dispersion}}=-s_{6}\sum_{n}^{N}\sum_{m}^{N}\frac{C_{6}}{R_{\mathrm{nm}}^{6}}\cdot f_{\mathrm{damping}}\text{.}$$

They make use of either system-independent (D2-type [26]), or system-specific (D3-type [27]) *C*_6_ coefficients, and (*s*_6_ and damping function) parameters refitted for the specific SQM methods at hand. The H terms are running over all possible donor (D)–hydrogen (H)–acceptor (A) combinations, and differ mainly in how they make use of geometric information to model the directionality of hydrogen-bonds. The full description of the arrangement of two molecular fragments requires 4 coordinates, for instance one distance (H…A), the ‘main’ hydrogen-bond angle (D–H…A, *θ*), a ‘secondary’ (H…A–R angle, *ϕ*) and a (H…A–R–R, *ψ*) torsion angle. But this requires a pre-definition of which electronegative atom is the donor and which one is the acceptor and if this distinction should be avoided, three more coordinates (distance, angle, torsion angle) are needed. A detailed overview of all technical details can be found in the above mentioned review [25], where we have also introduced a distinction between zeroth-, first-, second- and third-generation H terms based on how complete the geometric picture is as follows:

(2) $$E_{\mathrm{hydrogen}‐\mathrm{bond}}=g_{\mathrm{distance}}\cdot h_{\mathrm{orientation}}$$

(3) $$g_{\mathrm{distance}}^{0th/1st/2nd‐\mathrm{generation}}=f\left(r_{HA}\right)$$

(4) $$g_{\mathrm{distance}}^{3\mathrm{rd}‐\mathrm{generation}}=f\left(r_{DA}\right)$$

(5) $$h_{\mathrm{orientation}}^{0th‐\mathrm{generation}}=1$$

(6) $$h_{\mathrm{orientation}}^{1st‐\mathrm{generation}}=f\left(\theta \right)$$

(7) $$h_{\mathrm{orientation}}^{2nd‐\mathrm{generation}}=f\left(\theta ,\phi ,\psi \right)$$

(8) $$h_{\mathrm{orientation}}^{3nd‐\mathrm{generation}}=f\left(\theta ,\phi ,\phi 2,\psi ,\psi 2,r_{HX}\right)\text{.}$$

For zeroth-generation approaches, non-directional terms are added; within the first generation directional terms that depend only on the acceptor–hydrogen distance and the main (donor–hydrogen–acceptor) angle are introduced; within the second generation these terms depend also on secondary (base–donor/acceptor–hydrogen) angles and torsional (base–donor/acceptor–hydrogen) angles. For third-generation schemes (currently only PM6-DH +), the distinction between acceptor and donor atoms is skipped to allow for proton transfer processes and avoid several conceptual problems with adding MM-type terms to an electronic structure theory method. Third generation terms thus model hydrogen-bonds as an interaction between two electronegative atoms, which is smoothly switched on by the favorable placement of one (or more) hydrogen atom(s) in between them. This way, PM6-DH + needs only one fit parameter per electronegative element (O,N) to reach chemical accuracy in comparison to high-level CCSD(T)/CBS references for typical benchmark sets of non-covalently bound complexes. The main benefit of second-generation terms was the exclusion of unphysical interaction contributions which arise for first-generation terms [25,28,29], leading to substantial problems with geometry optimizations. One benefit of the SQM H terms in comparison to the earlier MM correction terms is their excellent transferability and the very low number of parameters needed for high accuracy. Also the DH2 scheme for instance is based on only 3 global and 5 method-specific atom-type based parameters which were optimized for OMx, PM6, AM1 and SCC-DFTB and lead to a very similar and excellent performance (again in comparison to high-level CCSD(T)/CBS references) for all investigated SQM methods. Further important developments were introduced by Foster and Sohlberg and Hobza and co-workers: In 2012 Foster and Sohlberg published a scheme for the self-consistent addition of charge-dependent terms (a problem most of all for PM6-DH2) [30]. Hobza and co-workers additionally included halogen-bond (X) terms, which work analogously to hydrogen-terms, but with an opposite sign, as standard SQM methods underestimate repulsion in halogen-bonds and thus deliver distances too short and interaction energies too high [31,32].

## 3 SQM methods, SQM-DH method development and benchmarking

### 3.1 SQM methods

Recent reviews on ‘pure’ SQM methods were published by Voityuk [33] and Thiel [34], the related density functional tight-binding (DFTB) approach was covered by Gaus et al. [35] with a special focus on organic and biomolecular system. Thiel [34] has published extensively on the investigation of dynamical effects on complicated electronic processes with his MRCI-SQM approach. His work highlights the fact that simulations of sufficiently large system size and simulation length are not possible with other QM methods. Voityuk especially emphasizes the importance of semi-empirical methods when screening electronic properties or analyzing trends rather than focusing on individual systems. Reviews related to the topic of describing non-covalent interactions with SQM methods have been published before by Foster and Sohlberg [36] (including a comparison to DFT methods), Korth [37] (focusing on hydrogen-bond correction terms), and Hobza [38,39] (concerning non-covalent interactions and accurately describing protein–ligand interactions).

### 3.2 SQM-DH method development

The first dispersion corrected SQM methods were developed independently of each other by Collignon et al. in 2006 (AM1-D) [40] and McNamara and Hillier [41] in 2007 (PM3-D, AM1-D). Tuttle and Thiel published a corresponding version of OMx in 2008 [42]. In 2009, Rezac et al. [43] published the first dispersion and hydrogen-bond corrected SQM method, PM6-DH, which was soon revised into PM6-DH2 by Korth et al. [44] A somewhat similar approach to add MM dedicated hydrogen-bond terms as a QM/MM interface term (in addition to the usual dispersions and repulsion terms) was pursued by Wang and Bryce in 2009 [45]. In 2010 Foster and Sohlberg published their AM1-FS1 model [30], which also makes use of both hydrogen-bond and dispersion terms. Korth continued after PM6-DH2 with focusing on conceptual improvements in his PM6-DH + approach [46], which allows for proton transfer reactions and uses only 2 fit parameters while not loosing accuracy in comparison to PM6-DH2. Rezac and Hobza continued after PM6-DH2 as well, with including also halogen-bond correction terms in their PM6-DH2X model [47] in 2011 (similar corrections were later on also applied to MM methods [48,49]). In 2012, Rezac and Hobza developed the PM6-D3H4 model with both improved dispersion corrections terms of D3-type and a new hydrogen-bond correction scheme of second-generation type, but with an increased robustness for geometry optimizations and molecular dynamics simulations [50]. Foster and Sohlberg continued their work with self-consistent, atomic charge dependent hydrogen-bond correction terms (which are usually added as post-SCF correction and thus lead to non-variational methods if partial charges are used) in 2012 [51]. In 2013, Stewart published the PM7 model [52], which includes dispersion and hydrogen-bond correction terms of mixed PM6-DH2/PM6-DH + type directly into the SQM fitting process. Together with Korth, Jensen and co-workers published PM6-D3H +, an updated PM6-DH + model with improved dispersion corrections of D3-type and a more robust (third-generation) H + term for an improved performance in geometry optimizations and molecular dynamics, in 2014 [53]. Later in 2014, Grimme, Bredow and co-workers published MSINDO-D3H + [54], using the more recent D3-type terms and the above mentioned improved implementation of the H + term for enhancing Bredow's MSINDO approach.

Apart from the above listed substantial contributions, a number of additional PM3-D and AM1-D models were published, for instance by Anikin et al. [55] in 2012, though methods like PM6-DH2 and PM6-DH + are readily available in MOPAC [56] from MOPAC2009 on. An important development related to SQM-DH type methods was the publication of corresponding GPU-enabled algorithms by Carvalho Maia et al. [57] in 2012, which allowed for a very impressive illustration of the capability of PM6-DH + to identify native protein structures out of large sets of decoy conformations.

### 3.3 SQM-DH method benchmarking using small biomolecular model systems

Extensive benchmarking was done for all SQM-DH methods already in the original papers and in a number of dedicated benchmark papers. Both the original and most benchmark studies have a strong focus on biomolecular systems and base their conclusions on benchmark sets of comparably small model systems: In 2011, Prenosil et al. have studied hydrogen-bond cooperativity effects with PM6-DH2 in comparison to MM, DFT and WFT methods [58]. Unlike MM methods, PM6-DH2 performs reasonable accurate in comparison to high-level Coupled Cluster data. In 2012 Rezac et al. investigated the performance of their newer PM6-DH2X and PM6-D3H4X models for a large number of halogenated systems [59]. In 2013 Hostas et al. in addition used OMx-D and PM7 to benchmark the performance of all these methods for non-covalent interactions including conformational changes [60]. Sedlak et al. finds an excellent price/performance ratio for enhanced SQM methods in a study on large dispersion-dominated biomolecular systems in 2013 [61]. In 2014, Li et al. published a benchmark with Hobza's BEGDB benchmark database using several SQM, SQM-DH, DFT, and symmetry-adapted perturbation theory (SAPT) methods [62]. They conclude that SQM-based methods are dramatically faster than DFT and SAPT methods (and thus readily available for large systems), but also that they are somewhat less accurate. No tested SQM-DH methods was found to be clearly superior with respect to the achieved accuracy; the authors do not discuss the conceptual advantages and disadvantages of the individual methods. Barberot et al. published a very interesting benchmark in 2014 [63], using their AlgoGen genetic algorithm approach for the extensive sampling of the conformational space of typical benchmark set systems to investigate the performance of the PM6-DH + model, for which several spurious minima are found.

### 3.4 SQM-DH method benchmarking using large biomolecular model systemsx

Few benchmark studies try to systematically evaluate SQM-DH methods for sets of realistically large model systems: In 2012, Mikulskis et al. investigated the performance of MM and SQM methods including (their own versions of) dispersion and hydrogen-bond corrections for protein–ligand interactions [64]. They highlight the importance of empirical corrections for SQM methods and suggest their AM1-DH version as a competitive alternative to MM/GBSA calculations. In 2013, Yilmazer and Korth studied the performance of PM6-DH + in comparison to DFT methods for several hundred systematically generated protein–ligand model systems from the PDBbind2007 benchmark set [65]. An excellent performance of the SQM-DH method in comparison to DFT-D data is found. In 2014, Korth and co-workers continued with comparing PM6-DH + and DFT methods to high-level WFT reference data for the smallest complexes of their previous study [66], giving further support to their earlier conclusions.

### 3.5 SQM-DH method benchmarking for interactions in water

A number of studies focus on the performance of (enhanced) SQM methods for describing interactions in water, including solvation and proton-transfer phenomena (with the latter one being accessible to SQM, but not standard MM methods): Bulo et al. published a benchmark of solute–water interactions using PM6-DH + and DFTB in a QM/MM setup in 2013 [67], focusing on the investigation of QM/MM setup parameters. In 2014, Marion et al. benchmarked the performance of amongst other methods OMx-D, PM6-DH2, PM6-DH +, PM6-D3H4 and PM7 for the interaction of water with hydrophobic groups [68], and arrived at a new model, PM3-PIF3 to describe systems in aqueous solutions. Work on constructing special models for proton-transfer in water (OM3n by Wu et al. in 2013 [69] or AM1-W and AM1PGG-W by Wang et al. in 2014 [70]) has shown that such models outperform ‘pure’ SQM methods, but consistent data for a comparison with SQM-DH methods is still missing.

### 3.6 Other

A series of papers by Merz and co-workers investigated the propagation of systematic and random errors in the computation of protein–ligand interaction energies and protein-folding including MM, SQM(-DH) and DFT data [71–74]. As expected, DFT was found to be the best choice, but interestingly some MM potentials outperformed some (non-enhanced) SQM methods, illustrating the need for empirical corrections. Truhlar and co-workers benchmarked amongst other methods PM7 in their papers on the development of their polarized molecular orbital (PMO) method [75–77] (with damped dispersion and p orbitals on H atoms for — also hydrogen–bond type–polarization effects, currently parameterized for H, C, N, O, and S), finding that PMO2 outperforms PM7 for instance on their set of atmospherically relevant compounds. Many benchmark studies do unfortunately not include SQM-DH models, but only AM1-D and PM3-D their work [78,79], accordingly finding a sub-optimal performance of SQM methods for hydrogen-bonded systems.

## 4 Applications

### 4.1 (Pre-)optimization, dynamical studies, structure refinement, conformational searches

Typical applications for SQM-DH methods include the fast optimization of molecular systems [80,81], especially for the evaluation of experimental data [82–85], the pre-optimization of biomolecular systems for subsequent higher-level computations [86,87], dynamical QM studies of biomolecular systems [88–92], and the use of SQM-DH methods as intermediate level for multilayer hybrid approaches, for instances within DFT-D/SQM-DH/MM computations [93], or even as QM method in dynamical QM/MM simulations [94]. An application which will likely become more and more important in the future is the SQM-DH based X-ray structure refinement [95]. If non-covalent interactions are essential, SQM-DH based refinement of structures might in general be a good idea [96]. Lupan et al. for instance found very good results for *α*-helical structures [97], also in comparison to DFT methods. Especially PM6-DH + proved valuable as a basis for the non-local optimization of molecular structures, for instance for screening the conformational space of the FGG tripeptide [98] or DNA quadruplex/molecule complexes [99].

### 4.2 Host/guest systems

The very good accuracy of SQM-DH methods for biomolecular interactions in comparison to high-level CCSD(T)/CBS references begs the question of whether these methods might not be good enough to replace DFT-D methods for the accurate computation of such interactions in medium sized systems. Accordingly many studies on host/guest or inclusion complexes were published over the last years [100–104], as well as on molecular tweezers [105], non-covalent complexation in general [106–110], and supramolecular chemistry [111,112], most of them with very promising results at SQM-DH level. Muddana and Gilson presented a study on 29 CB7 host-guest systems with PM6-DH +/COSMO based on their ‘minima mining’ (M2) approach for predicting binding affinities and found good agreement with experiment [113]. Their blind prediction of 14 CB7 binding affinities within the SAMPL4 challenge [114] was less successful due to two unclear outliers possibly related to technical problems [115].

### 4.3 Virtual drug design

SQM-DH methods are already and very successfully used as an accurate but fast tool for rational drug design and optimization (as DFT-D methods are usually not applicable to a large number of large enough model systems): An excellent illustration of the opportunities offered by SQM-DH methods was given by an impressive study from Avila Salas et al. in 2012 [116]. The interaction energies of 4 drug molecules with 8 polyamidoamine dendrimer fragments were computed from overall 320 million configurations of about 30 to 170 heavy atoms with PM6-DH + on a compute grid. The SQM-DH results show an excellent correlation with experiment (*R*^2^ = 0.9), especially in comparison to MM data (*R*^2^ = 0.75). Benson et al. [118] on the other hand used PM6-DH2 to predict the enthalpic part of the SAMPL3 challenge [117] trypsin/fragment binding affinities in combination with different solvation models [118]. They emphasize the importance of (multiple) docking poses and the influence of the solvation model, so that at least in their approach SQM-DH methods offer no benefits over purely empirical scoring functions. Hobza and co-workers scored 22 HIV-1 ligands with PM6-DH2/SMD (and MM-based entropy terms) [119], resulting in a ‘dramatic’ improvement over the conventional DOCK procedure. The authors emphasize that their scheme is free of system-specific parameters and thus readily available also for other protein/ligand systems. In 2011 they continued with scoring 15 structurally diverse CDK2 inhibitors with PM6-DH2/COSMO and again found very good agreement with experiment [120]. Later on they investigated SmCB1 inhibitors and obtained results in close agreement with DFT-D data [121,122]. Very recently Hobza and co-workers investigated malonate-based inhibitors of mammalian serine racemase [123] and decided to add a new repulsion correction to their PM6-D3H4X/COSMO approach.

SQM-DH methods are now more and more often used for QM-based scoring [124,125], QM/MM-based scoring [126,127] (sometimes unsuccessfully though still in good agreement with DFT [128]) and the *in silico* design of biologically active compounds [129]. Two especially interesting studies need to be highlighted: In 2012 Stigliani et al. presented a docking study based on Autodock Vina structures re-ranked with PM6-DH2/COSMO, clearly improving the results [130]. In 2014 Ucisik et al. published a very interesting study on predicting protein–ligand binding affinities using Monte Carlo estimates of the configuration integrals based on SQM-DH microstate energies using different implicit solvation models [131]. A especially good correlation with experiment is found for PM6-DH2-based estimates. For the future, it seems likely that SQM-DH methods will turn to be particularly useful for the *de novo* design and optimization of functional biomacromolecules, a task for which MM methods are not accurate enough and DFT-D methods are not fast enough. First attempts include for instance the screening of peptides with anti-breast-cancer properties at different theoretical levels [132], the computation of DNA/zinc–finger–protein interactions [133], or the investigation of complete nucleic acids building blocks [134]. Similarly challenging are the interactions of organic molecules in the solid state and in solution [135].

### 4.4 Materials science

Turning from life science to materials science applications, a large number of ‘nano-systems’ including graphite, graphene, fullerenes, nano tubes, DNA bases and combinations thereof were successfully investigated with SQM-DH methods [136–149]. The low computational cost of these methods make them especially useful also for investigating molecular self-assembly [150–153], molecular switches [154] and for the screening of thousands of compounds for methane storage [155–157], as well as hydrogen storage [158]. To mention just one study in the field of materials science in more detail, Vincent and Hillier found that PM6-DH2 predicts adsorption energies of molecules on graphene with an accuracy comparably to DFT-D and in good agreement with experiment [159]. Conti and Cecchini find PM6-DH + to be the most efficient method for the calculation of small molecule adsorption energies on graphene in comparison to FF, other SQM and DFT approaches. They observe almost chemical accuracy for PM6-DH + results in comparison to temperature-programmed desorption experiments and present first tests for the rational design of improved graphene surfactants [160].

Also many other studies both in life and materials science found the performance of SQM-DH methods similar to DFT-D [84,91,92,97,122,143,144,153,161–166], sometimes even when aiming for high accuracy in complicated cases (like for instance the interaction of DNA bases with Li@C60 [167]), but a number of studies (often relying on the older AM1-D or PM3-D models) observe a lower quality of SQM-DH results [168–170].

The equally good performance of DH models for problems both in life and materials science illustrates the robustness of the general approach and is of high interest for inter-disciplinary research for instance on bio-nano structures and (bio-)organic/inorganic hybrid materials.

### 4.5 Other

More exotic applications include chiral discrimination [171] or piezoelectric effects of applied electric fields on hydrogen-bond interactions [172]. In 2013, Amorim Madeira et al. presented a very interesting application of PM6-DH +, computing protonation sites and proton affinities of amino acids with the goal of solving mass spectrometry problems [173]. Another especially interesting study was presented by Preiss and Saleh in 2013 [174], in which PM6-DH +-derived descriptors are used to estimate the glass transition temperature of 209 molecular liquids. Finally, Rappoport et al. need to be mentioned, who have developed a heuristic approaches to estimate kinetic effects in complex chemical reaction networks [175], using PM7 (including DH terms) for structure optimization and reaction thermodynamics.

## 5 Conclusions and outlook

The overview given above clearly illustrates the substantial contribution of DH corrections to the revival of SQM methods in general by opening up the fields of life and (some parts of) materials science for their application. The most important message seems to us: Similar to D-type corrections for DFT methods, DH-type corrections for SQM methods should be used whenever non-covalent interactions are essential for the problem at hand; the actual version of the correction is less (though not un-)important. As for MM methods, the importance of dedicated hydrogen-bond terms [176] and the use of a complete geometric description is questioned sometimes [177], but these negative conclusions are almost exclusively based on data from simple potential functions, while the opposite is found when complete models are investigated [28,29,178].

Almost all SQM-DH methods reach an excellent accuracy for non-covalent interactions in comparison to DFT methods, but some of them offer conceptual advantages over others (see Introduction section). The biggest problem seems to be the existence of artifacts on the potential hypersurfaces of most models, like oscillations during geometry optimization of small, highly-symmetric systems with for instance PM6-DH +. Hobza claims to have solved this with his latest approach (but has sacrificed the complete geometrical description for this), Korth, Jensen and co-workers have released a new implementation of the DH + model (also with updated D3 dispersion terms) to address this problem. To the best of our knowledge, only PM6-DH2, PM6-DH + and PM7 (including mixed DH +/DH2-type terms) are publicly available (through MOPAC [56] from MOPAC2009 on) at the time of writing, though also the improved D3H + code by Kromann et al. is freely available on GITHUB [179].

From the methodological point of view, several further improvements of SQM-DH methods seem possible, as almost no part can be considered perfect (see our previous review for details [25]). One should on the other hand mention, that the theoretically most stringent way to deal with hydrogen-bonds in SQM methods is likely the inclusion of orthogonalization corrections, as it is done within the OMx methods by Thiel and co-workers [180–183]. Currently, these methods are parameterized only for first-row elements, but there are many activities in Thiel's group to make OMx methods more versatile and even more accurate. Nevertheless, SQM-DH methods are clearly a valuable addition to the method zoo of computational chemistry. Other important developments in the field include substantially improved SQM models like the above mentioned PMO by Truhlar and co-workers, as well as models derived from higher-level theory approaches like the one suggested by Laikov in 2011 [184]. There is also the recently developed AM1/d-CB1 method (including some d-orbitals and new core-core repulsion terms) [185,186], but benchmark data in comparison to SQM-DH is not yet available.

Noteworthy is also the further development of minimal QM models (especially HF-3c by Sure and Grimme [187]), which are somewhat slower, but generally more robust than SQM-DH methods and thus capable of filling the gap between SQM and DFT methods in terms of cost and accuracy. In a recent perspective Grimme, Bredow and co-workers highlight several variants of low-cost quantum chemical methods (SQM-DH, HF-3c, SCC-DFTB) as pre-screening tools and for computing thermostatistical corrections. The authors conclude that due to their improved accuracy and application range ‘the future for low-cost quantum chemical methods for investigating NCIs in all of their aspects seems bright’ [54]. There is not much to add to this from our side.
